# Applications of Blockchain-Based Technology for Healthcare Devices Post-market Surveillance

**DOI:** 10.7759/cureus.57881

**Published:** 2024-04-08

**Authors:** Swarna Muniappan, Madhan Jeyaraman, Sankalp Yadav, Naveen Jeyaraman, Sathish Muthu, Swaminathan Ramasubramanian, Bishnu P Patro

**Affiliations:** 1 Electronics and Communication Engineering, Dr MGR Educational and Research Institute, Chennai, IND; 2 Clinical Research, Viriginia Tech India, Dr MGR Educational and Research Institute, Chennai, IND; 3 Orthopaedics, ACS Medical College and Hospital, Dr MGR Educational and Research Institute, Chennai, IND; 4 Department of Orthopaedics, Orthopaedic Research Group, Coimbatore, IND; 5 Medicine, Shri Madan Lal Khurana Chest Clinic, New Delhi, IND; 6 Department of Orthopaedics, Government Karur Medical College, Karur, IND; 7 Department of Biotechnology, Faculty of Engineering, Karpagam Academy of Higher Education, Coimbatore, IND; 8 Orthopaedics, Government Medical College, Omandurar Government Estate, Chennai, IND; 9 Orthopaedics, All India Institute of Medical Sciences, Bhubaneswar, IND

**Keywords:** artificial intelligence, internet of things, medical device, block chain technology, post-marketing surveillance, blockchain

## Abstract

The volume of data analysis for medical device post-market surveillance (PMS) has increased dramatically in recent years. It is the more stringent and intricate regulatory criteria of the health authorities that are meant to improve the medical device safety review. As regulators scrutinize device safety more closely, proactive approaches to PMS processes are becoming crucial. To solve some of the issues brought on by this shifting regulatory landscape, new technologies have been investigated. This study envisages the technical features of blockchain technology (BCT) and its role in enhancing the PMS for medical devices. To address the aforementioned challenges, our model involves the establishment of a secure, permissioned blockchain for PMS data management, utilizing a proof-of-authority consensus mechanism. This blockchain framework will exclusively permit a carefully vetted and designated set of participants to validate transactions and record them in the PMS data ledger. The utilization of BCT holds the potential to introduce enhanced efficiency and provide several advantages to the various stakeholders involved in the PMS procedure, including its potential to support emerging regulatory efforts.

## Introduction

The system for monitoring medical devices after they're released into the market, known as post-market surveillance (PMS), has been facing an exponential increase in data volume in recent times. This surge in data is largely due to the tightened regulatory standards set by health authorities, aimed at improving the safety assessments of medical devices [1,2]. These authorities are moving towards establishing a system where safety information is exchanged swiftly, reliably, and efficiently. The ultimate goal is to ensure that any safety risks associated with medical devices are detected promptly and that necessary corrective actions are taken without delay [3,4]. As a result of the increasing rigor in safety checks, there's a significant shift towards enhancing PMS systems to be more proactive. Industry stakeholders, including those responsible for ensuring the safety of medical devices, are fully aware of the challenges posed by the evolving regulatory environment. They acknowledge the urgent need to overcome these challenges and to close the existing gaps in the PMS processes [5].

Parallel to developments in other sectors of the medical device industry, there's a growing interest in the application of artificial intelligence (AI) technologies to revolutionize traditional, reactive PMS systems [6,7]. Among the array of technologies being explored, blockchain technology (BCT) has garnered significant attention over the past two years. Blockchain can essentially be described as a distributed ledger or database that facilitates data processing through a network of interconnected nodes [8]. Unlike conventional data-sharing methods that rely on a central authority for data management, blockchain offers a decentralized approach. This decentralization enables direct interactions among users, eliminating the need for third-party intermediaries. Traditionally, transactions between two parties (users A and B) required verification by a third party (user C), which could be a government agency or a healthcare regulatory body. However, with the advent of blockchain, this intermediary's role becomes obsolete. BCT empowers users to encode and record diverse types of transactions in a secure, immutable, and distributed ledger [9]. This innovation paves the way for transactional models that bypass traditional intermediaries, offering new levels of efficiency and security. The regulatory landscape for PMS is complex, marked by a need for robust data integrity, transparency, and traceability, alongside the requirement for rapid response mechanisms to adverse events. Blockchain technology, characterized by its decentralized, transparent, and immutable ledger system, offers a transformative solution to these challenges.

In this article, we delve into the current challenges faced by the PMS system, highlighting the complexities of managing increased data volumes and regulatory demands. We explore how blockchain technology can be harnessed to enhance PMS, discussing the strategies for its implementation and examining the potential it holds for transforming safety surveillance of medical devices. Additionally, we address the limitations of BCT in the context of PMS, providing a balanced perspective on its applicability and impact. Through this discussion, we aim to offer insights into the future of medical device safety and the role of innovative technologies in shaping it.

## Technical report

Blockchain technology

This article aims to examine the various technical facets of BCT and its potential to bolster ongoing efforts to enhance the PMS system for medical devices. A blockchain is a distributed and decentralized digital ledger, often publicly accessible, designed to record transactions across multiple computers [9]. Its fundamental feature is ensuring that once a record is added, it cannot be altered retroactively without modifying all subsequent blocks in the chain. Blockchain is an open-source technology that facilitates secure communication for tracking previous actions related to decentralized patient records [10,11]. For instance, it enables the effective sharing of safety data while upholding the principles of integrity, privacy, and accessibility [11]. This innovative approach has the potential to make substantial volumes of anonymized PMS data from diverse sources accessible, including unsolicited reports, medical device registries, and unconventional data sources [12-14].

**Figure 1:**
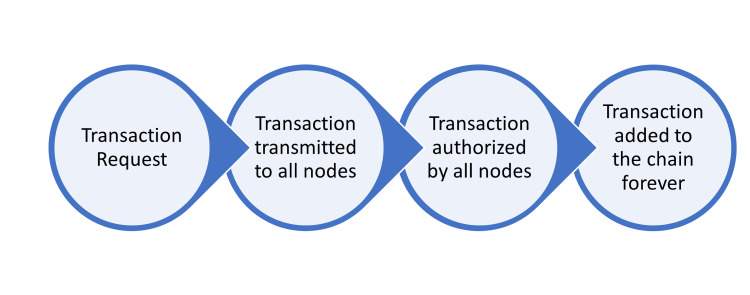
Illustration of process workflow in the blockchain technology Picture courtesy: Dr. Sankalp Yadav and Swarna Muniappan

With BCT, several participants in a network can share a single ledger that they can all rely on to be accurate. Every new transaction or piece of data is added to a "block," which is made up of all the previous blocks' hashes, linking them all together to form a chain of blocks known as a blockchain [3]. By continuously chaining hash-based proof-of-work transactions together, the network timestamps them and creates a record that cannot be changed without repeating the proof-of-work. These transactions are recorded and can be used to support payments and currencies, as well as provide safety information. Every new transaction on this network must be verified by a node in order to guarantee its completeness. Every block added to a blockchain makes each transaction in that block more unchangeable since every node in the network verifies it. The blockchain process's workflow is depicted in Figure 1.

On a public blockchain, individuals have the capability to examine the ledger, and anyone can also contribute to and authenticate a block of transactions [9]. Conversely, in the context of private blockchains, it typically allows anyone on the internet to observe transaction blocks, but only designated individuals within organizations possess the authority to add and validate these blocks [10]. In the case of consortium blockchains, a specific category of groups within a company, such as banks, is granted the privilege to verify and add transactions, and the accessibility of the ledger can either be extended to a broader audience or confined to the chosen group [15-17].

Difficulties in providing sufficient PMS for medical equipment

It has been determined that the following problems will make it difficult to perform sufficient PMS on medical devices.

Security and Data Interchange

The rising integration of new technologies with medical equipment raises concern about potential PMS data breaches, which must be prevented. It is essential to establish a secure environment for the swift and accurate sharing of data with authorized parties. One of the primary challenges in the safety signal detection process for medical devices is the timely exchange of PMS data between various sources and documents [7,18]. Recent public health crises have underscored the consequences of delayed identification of safety issues associated with promoted medical equipment.

Trackability of Medical Equipment

A robust PMS system necessitates the identification of the root cause behind adverse events. The examination of a sample of medical devices is essential for pinpointing the failure mode associated with an incident, but often this step falls short in determining the underlying cause [19,20].

Imitation

A recognized risk to patient safety is the counterfeiting of medical equipment. Increasing regulatory demands for further details regarding the provenance of medical devices have been made in an effort to solve this problem [21]. It is necessary to swiftly locate any medical device on the market in order to carry out safety-related regulatory measures, and this calls for the use of a Unique Device Identifier (UDI) [22].

Harmonization

The absence of uniformity and standardization among PMS data sources represents a significant challenge in the process of detecting safety signals for medical devices [18]. Each PMS data source contains unique content and employs different data storage methods. Two primary categories of medical device PMS data sources are spontaneous reporting systems and medical device registries, each with its own set of advantages and disadvantages.

Spontaneous Reporting Systems (SRS)

SRS are dynamic systems designed to capture patient injuries and product issues reported directly or through published articles [23]. Reports are sourced from patients, healthcare professionals, regulatory bodies, and manufacturers. The structure of SRS relies on establishing connections between events and specific medical devices. The extensive datasets collected are typically processed centrally, often within a database or repository, facilitating accessibility for analysis.

However, the absence of a globally harmonized standard dataset for reporting poses challenges in integrating data from diverse databases [24,25]. Moreover, a substantial portion of investigative findings remain inconclusive, and there is a bias towards reporting well-known adverse events or product issues related to medical devices, often influenced by social media or media coverage. Additionally, underreporting and over-reporting issues arise when medical devices undergo inadequate testing or inspection procedures [26].

Medical Device Registries

A medical device registry is a structured system with the primary objective of enhancing awareness of the role that medical devices play in elevating the quality of patient care consistently [27]. It systematically collects relevant data, evaluates significant outcomes, and comprehensively addresses the population's exposure to specific medical devices on a broad scale, ranging from global to national, regional, and healthcare system levels [28]. These registries typically contain essential information such as medical device details, diagnoses, prescriptions, medical histories, and procedures [29].

Unlike spontaneous reports, medical device registries are not limited to individuals who have experienced adverse effects or difficulties with medical devices [28]. Consequently, the data obtained from medical device registries offers distinct advantages that can complement more traditional PMS data sources, such as SRS. This includes the ability to conduct active PMS and confirmatory investigations. While the utilization of medical device registries offers numerous benefits, it is essential to acknowledge their associated limitations and drawbacks as well.

The registry's features may differ between nations due to variances in the consistency, granularity, and quality of the data, as well as in the length of the longitudinal follow-up, attrition rates, data privacy rules, legislation, and degree of information interchange [30,31]. A delay in the collection and consolidation of PMS data from foreign registries could result from the absence of standards among the various registries, which would ultimately delay the verification of safety signals. Device outcome data from the registry cannot be analyzed properly if a standardized UDI and nomenclature codes are not used. User errors are a major cause of unfavorable incidents involving medical equipment. To guarantee the safe handling of medical devices, it is imperative to develop suitable risk mitigation measures, primarily training.

BCT for PMS

**Figure 2:**
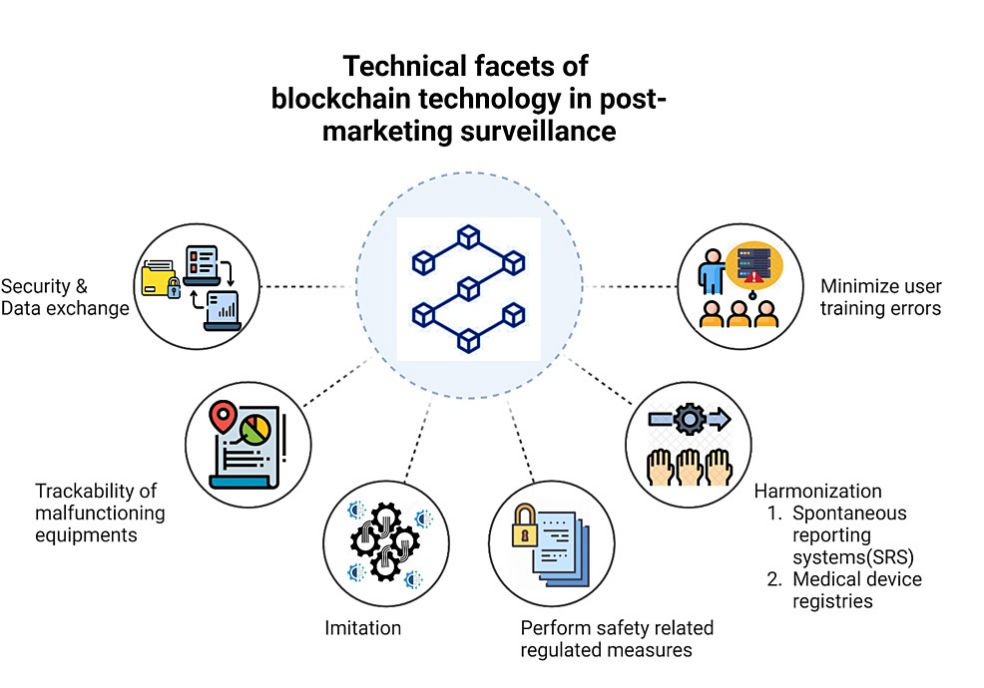
Technical facets of blockchain technology in improving the current limitations of traditional post-marketing surveillance Picture courtesy: Dr. Madhan Jeyaraman and Dr. Sankalp Yadav

Prospects for utilizing BCT to resolve issues with the PMS system in medical devices, either as a private permissioned blockchain or as a public permission-less blockchain, show promise. It might deal with a number of the issues that have been previously stated, as shown in Figure 2.

Trackability of Medical Equipment

The global adoption of UDI may very well be supported by BCT. Blockchain makes it possible to record production data as well as continuing use or maintenance data. Its unchangeable and dependable workflow will offer total traceability to medical device producers and proof of any safety concern related to that particular medical equipment. With the implementation of the European Union (EU) Medical Device Regulation's (MDR) extra traceability criteria, this kind of technology is becoming more and more important. According to the new rule, every product packaging must have a UDI that is readable by both computers and humans. Annex VI of the MDR addresses the utilization of data collection and automatic identification technologies, such as QR codes or bar codes, which have the potential for integration with BCT [7,32]. This integration envisions a scenario where a single bar code not only contains machine-readable data but also provides access to a blockchain traceability system.

Within this system, every stage of the supply chain and every interaction with the medical equipment is meticulously recorded by the blockchain traceability tool. Access to this blockchain would be extended to all economic operators involved with the medical equipment, allowing them to scrutinize the device's interactions comprehensively [7]. This approach facilitates efficient access to critical data for health authorities. Moreover, end-users can independently verify the authenticity of a medical device by scanning the blockchain, cross-referencing its data with the UDI database, and tracing its journey through the supply chain [33,34].

The regulation stipulates that "manufacturers must periodically verify the accuracy of all data relevant to the devices they have placed on the market" and implement "appropriate methods for validating the provided data" [35]. A blockchain-driven repository offers specific functionalities that align with the requirements of regulatory authorities.

Antitrust Actions and Fake Goods

The widespread implementation of UDI and BCT has the potential to significantly improve the efficiency of regulatory actions by enabling the comprehensive tracking of all medical products on the market and facilitating their swift and effective removal when necessary [36]. BCT possesses the capability to monitor every stage of the medical equipment supply chain by meticulously tracking each transaction [37]. This integration of BCT can bolster data security, elevate the traceability of medical devices throughout the entire supply chain, and contribute to the detection and prevention of medical device fraud [38,39].

PMS Data Security, Standardization, and Interchange

Blockchain transactions are transparent, trustworthy, and secure records that cannot be duplicated or tampered with, making them valuable for detecting fraudulent activities [40]. BCT holds the potential to revolutionize the proactive approach to PMS data collection, encompassing spontaneous reports, registries, and nonstandard data sources [41]. It enables direct data acquisition without relying on the active reporting of adverse events. For instance, when a patient's information is registered in a medical device registry or a healthcare provider records a device-related adverse event in an electronic health record, this data can automatically become part of a shared block of information, eliminating the need for conscious incident reporting.

This innovative approach has the promise of significantly increasing the quantity of post-market data collected with minimal human intervention, ultimately enhancing the quality of the gathered PMS data [42]. Blockchain provides a decentralized and secure framework for sharing various types of safety information, making it less susceptible to cyberattacks [42]. New storage solutions that leverage BCT offer permanent and secure storage of medical device PMS data while maintaining efficiency and cost-effectiveness. The integration of blockchain in PMS has the potential to standardize data formats and content from various sources, enhance the security and efficiency of data exchange, ensure data integrity and transparency, and reduce the need for human intervention at each stage of data creation and retrieval [7,43]. The distributed nature of blockchain and the requirement for consensus to alter the chain enhance data integrity.

While public blockchains may lack privacy as data is open for inspection by all participants (albeit pseudonymized), "private" blockchains, restricted to select stakeholders, can provide the necessary privacy measures. These private blockchains can facilitate the exchange of PMS data among various stakeholders involved in the medical device safety evaluation process [44,45].

User Mistakes

BCT has the potential to expedite the identification of safety issues related to software devices caused by user error [46]. It can promptly inform the manufacturer about the nature of the fault and the specific training required for its resolution.

Strategic recommendations

A systematic implementation plan with clear goals, timelines, roles, and responsibilities is crucial for the successful integration of the private-permissioned blockchain into the PMS of medical devices. To fund this initiative, a consortium comprising various PMS stakeholders, including health authorities, manufacturers, and hospitals, should collectively provide financial support, with International Medical Device Regulatory Forum (IMDRF) assuming the role of coordinator [47]. Each member is required to pay annual fees to secure the necessary resources for implementing and operating the new PMS system for medical devices, built on the private, permissioned blockchain. This framework also has the potential for future expansion to include the Eudamed database.

Phase I: Standardization

IMDRF is a coalition of medical device regulators from different nations that have voluntarily joined together to harmonize regulatory requirements for medical products that vary across countries. This consortium includes regulatory bodies such as the Russian Ministry of Health, Singapore's HSA, Australia's TGA, Brazil's ANVISA, Health Canada, China FDA, the European Commission, Japanese PMDA and MHLW, and the US FDA [47-49]. IMDRF is embarking on an initiative to standardize PMS requirements across various jurisdictions. IMDRF will oversee the standardization efforts necessary for the implementation of the new system, ensuring that PMS data is consistently recorded in the blockchain system mentioned in the "Expert Review of Medical Devices" [47].

To reach a consensus on critical aspects like identifying PMS data sources, criteria for reporting adverse events, coding dictionaries for adverse events, and global device identification systems to be used in the new private permissioned blockchain, IMDRF will engage in negotiations with the diverse group of PMS stakeholders, which includes health authorities, hospitals, and manufacturers [7].

Phase II: Development of Permissioned Blockchain for Private Data and a New Global PMS Database

Once the global PMS requirements and the new global adverse event reporting dataset have been established, IMDRF will initiate collaboration with a technology partner to develop the software for the upcoming PMS global database. This software will be built upon a new private permissioned blockchain with a proof-of-authority mechanism, ensuring the verification of every PMS data transaction [7]. To facilitate the use and participation in the new global PMS database, a governance framework will need to be created. All stakeholders, including health authorities, manufacturers, hospitals, and others, will need to reach a consensus and publicly disclose the regulations governing various aspects of the database. This includes rules related to accessibility, confidentiality, oversight, and regulatory reporting, as well as the agreements governing the permissioned blockchain for managing private data [50].

Phase III: Regional Pilot Program (US)

Following the consensus on global PMS standards, IMDRF will initiate a pilot program in the United States to implement the new private-permissioned blockchain system. In this endeavor, manufacturers will play a pivotal role in ensuring medical device traceability through blockchain by integrating BCT into their supply chain management processes [7]. Furthermore, they will be responsible for transitioning their existing PMS data sources, such as SRS and registries, to the blockchain platform.

As part of the pilot program, hospitals and health authorities participating in the initiative will need to commit to using blockchain for PMS data exchange. IMDRF will lead this pilot project, involving three US-based medical device companies, the FDA as a health authority, and three US hospitals. The primary objectives of this pilot are to demonstrate how blockchain can connect various systems and administrations to track a shared dataset containing patient data and product traceability information [7]. Additionally, it aims to showcase how BCT can potentially enhance the PMS of medical devices by expediting the process of notifying the supply chain about a medical device recall and facilitating the distribution of PMS data among stakeholders.

To facilitate the pilot, IMDRF will need to provide a technology partner and a consulting group. The technology partner will supply the PMS software, using the standardized reporting dataset established in Phase I, and will employ the blockchain infrastructure for verifying data transactions [7]. The consulting group will offer support, training, and oversight to pilot participants, ensuring accurate and comprehensive data recording throughout the process. Lessons learned from the pilot will be shared with all IMDRF members.

Phase IV: Global Pilot Program

Following the successful completion of the US pilot, a second pilot program will be launched on a global scale. IMDRF will once again play a crucial role in supporting this second pilot, which will involve nine medical device manufacturers (three from the US, three from the EU, and three from China), nine hospitals (three from the US, three from the EU, and three from China), and three health agencies (the FDA, European Commission, and China FDA) [51,52]. The primary objectives of this second pilot are to address unresolved issues and questions left by the US pilot and demonstrate how blockchain can connect various systems and administrations globally to track a shared dataset containing patient data and product traceability information while accommodating diverse local data privacy regulations. Additionally, it aims to illustrate how BCT could potentially enhance the PMS of medical devices on a global scale.

To facilitate the second pilot, IMDRF will need to provide a technology partner and a consulting group. These partners and consultants will collaborate with the manufacturers, hospitals, and national health authorities to supply the necessary tools, guidance, and support [51]. Given the global context, they will also play a crucial role in bridging any cultural gaps that may arise during the adoption of this new technology, such as language barriers, regional variations in technology, and the need to ensure PMS data confidentiality. The timeframe for the pilot is subject to adjustments and extensions. This flexibility will allow for modifications if any defects or limitations are identified during or after the pilot, or if additional areas of concern emerge to ensure the successful global adoption of the blockchain-based system. Lessons learned from the pilot will be shared with all IMDRF members.

Phase V: Global Blockchain Regulations

To ensure the successful implementation of the blockchain system by the globally agreed-upon GO-LIVE date, each local health authority will collaborate under the coordination of IMDRF [7]. They will develop and release local regulations and guidelines tailored to the specific needs of local hospitals and manufacturers. These regulations and guidelines will be established following the successful conclusion of the global pilot. The provided rules and guidelines will encompass instructions on how to manage data privacy, taking into account the pertinent local confidentiality laws. Additionally, the materials will outline the transition period for the worldwide implementation of the blockchain-based system, ensuring a smooth transition for all stakeholders involved.

Phase VI: Transitional Period

During the two-year transition period, PMS stakeholders will work in collaboration with the appropriate technology partner and consulting group (if required) to ensure a successful GO-LIVE date. The new private PMS data-permissioned blockchain will replace existing systems like SRS and medical registries [7]. Within this two-year window, all hospitals, manufacturers, and health authorities will have the opportunity to migrate to the new blockchain system. To facilitate this transition, local health authorities will provide local PMS stakeholders with technology support and training, ensuring that they are well-prepared to deploy the new blockchain system by the predetermined deadline.

Phase VII: Global Launch

After the GO-LIVE date and the completion of the two-year transition period, a dedicated team within the IMDRF organization will monitor the usage of the new system for any potential issues [7]. This team will provide training and technology support as necessary. Local health authorities will play a key role in ensuring compliance with the new private PMS data-permissioned blockchain regulations. They will conduct regular inspections of the PMS systems of various local stakeholders to verify that they are effectively utilizing the technology and adhering to the established regulations.

## Discussion

Limitations of BCT

In order to ensure the successful incorporation of BCT into the PMS process for medical devices, it is crucial to understand the challenges associated with this innovative technology.

Data Security and Privacy

BCT offers enhanced security by eliminating the need for third-party intermediaries in safety data transactions. However, it introduces certain privacy and security concerns due to its transparent nature, where the entire user community validates records within the blockchain architecture as opposed to a single external entity [5]. This lack of data privacy arises because every node can view the data transmitted by other nodes. In scenarios where a patient needs to designate a representative to access their information in emergencies, there is a risk that this representative may grant access to others, posing significant data privacy and security challenges. Another option to address these concerns is to implement robust security measures for the data, but this could impede data transfer across blocks and restrict access. Additionally, blockchain systems are vulnerable to a security breach known as a "51% attack," where a group of miners controlling over 50% of the nodes collaborates to manipulate the blockchain's contents [53]. This control allows them to halt new transactions, potentially disrupting the network, as evidenced by recent incidents affecting multiple coins. Furthermore, certain patient records may contain sensitive information that is unsuitable for inclusion on a blockchain, presenting additional complexities when using blockchain for PMS. To overcome these challenges, we propose the implementation of a private PMS data-permissioned blockchain with a proof-of-authority consensus mechanism [7,54-57]. This approach restricts the validation and addition of transactions to a small, vetted group of approved parties, addressing the aforementioned difficulties.

As an alternative, we may provide a trustworthy environment for decision-making, such as MedRec, a blockchain-based system that allows patients and medical practitioners to be authorized by manufacturers or health authorities to be new users of the private blockchain, safeguard, and recognize the PMS community members in charge of authorizing modifications and managing information sharing amongst the various parties involved [58,59]. Members of the PMS community now have the capability to contribute additional records associated with specific patients, and patients themselves can consent to sharing their records with other parties. All interactions among users and stakeholders prioritize utility, resulting in a single database where changes to patients' medical histories can be easily accessed. This concept holds the potential to enhance control over PMS data sharing for stakeholders and address data reliability issues, a prominent obstacle in PMS data exchange.

Another viable alternative involves ensuring complete data privacy for participants during PMS data transactions while maintaining transaction authenticity. However, in our private blockchain, some users may also be producers of medical equipment. In the conventional blockchain setup, participants could potentially access sensitive information about their competitors. To address this concern, our private PMS blockchain should implement a zero-knowledge proof algorithm method [60]. This method ensures that competitors cannot view transaction data belonging to their rivals while allowing transactions to be verified, thus eliminating the possibility of competitors gaining access to sensitive information about each other.

Oversee Data Retention

Effective management of data storage capacity poses a significant challenge. The traditional web and its associated data storage methods are vulnerable to data loss. In contrast, blockchain offers a decentralized data storage solution that is more resilient. BCT is primarily designed for tracking and executing data transactions. However, PMS generates substantial data volumes, necessitating frequent data archiving. Every node in the blockchain network must access the extensive PMS data stored in the blockchain, requiring substantial storage capacity [61]. The speed of identifying and editing events can be hindered by the proliferation of PMS databases, which is problematic for situations where rapidity is crucial in PMS data transactions. To address this scalability issue, a blockchain solution should offer ample storage capacity while ensuring permanent retention of PMS data, all while maintaining fast performance and cost-effectiveness [62]. For instance, this solution could involve the development of a platform that allows users to store PMS data on a blockchain indefinitely without compromising speed or affordability.

Interoperability Issues

The design of PMS databases differs significantly from that of BCT, which is characterized by its distributed and decentralized nature. PMS databases are typically offline, centralized, and local in their structure. To effectively implement BCT in the context of PMS, a functional PMS database that can facilitate communication among various PMS stakeholders needs to be established [63]. One of the primary challenges in implementing BCT for PMS is that many international regulations still require central health authorities to govern PMS data exchanges with strict adherence to data privacy requirements. To address these challenges, a solution could involve the adoption of a private PMS-permissioned blockchain with a proof-of-authority consensus mechanism [50,64,65]. This approach would limit transaction validation and ledger updating to a small group of authorized and audited participants, including manufacturers, reporting facilities, and health authorities. To ensure the successful deployment of BCT, new international guidelines may need to be developed, and existing PMS data sources would need to transition to the new private PMS-permissioned blockchain system.

Standardization Issues

To enhance the standardization of secure PMS data sharing, the integration of BCT is a promising approach. However, it's important to note that BCT is still in its early stages, which may pose challenges when attempting to standardize its use in the context of medical device PMS. To address this, international health authorities should take the lead in establishing global standards for evaluating shared data concerning its volume, type, and structure in blockchain applications within the PMS domain [53,66,67]. In cases where there is a lack of harmonized data sets, individual nations or regions may create separate blocks with distinct data sets. It's crucial to emphasize that for blockchain applications to deliver value in PMS, several prerequisites must be met. This includes ensuring that existing PMS data exchange platforms are fully interconnected and compliant with all relevant business regulations, which should be automatically enforced to guarantee the effectiveness of blockchain implementations [68].

Behavioral Issues

In addition to the technical challenges previously discussed, BCT is still in its early stages, and it faces behavioral hurdles related to cultural shifts. While the medical device industry is gradually transitioning toward digitization, there is significant work ahead before traditional administrative PMS data exchange tools can be entirely replaced by BCT, which has yet to establish a proven track record in PMS data exchange [69,70]. Effectively transitioning medical professionals, patients, manufacturers, and health authorities away from paper records and toward technology will require patience and comprehensive training efforts. Currently, both the technology itself and the associated legal frameworks are met with skepticism due to their relatively low adoption rates in the healthcare sector as a whole. To address these challenges, stakeholders involved in the PMS process for medical devices should offer educational resources aimed at sharing the advantages and disadvantages of BCT in the medical device sector [71-73]. This proactive approach can help promote understanding and acceptance of blockchain within the industry.

Capital Investment

Collecting data on the blockchain and building the required number of blocks to ensure its immutability can be expensive due to a shortage of skilled professionals capable of developing blockchain infrastructure [43,66]. Consequently, there may be a need to accelerate transaction processing time, measured in the number of transactions per second, as PMS data exchanges require swift completion to ensure early detection of safety concerns. This might entail paying additional transactional fees, which can become costly depending on the accessibility of the network. It is essential to thoroughly evaluate the cost implications of integrating this new technology into the PMS system. Some hospitals still lack comprehensive computerization, which hinders their ability to exchange data with others, especially if they are not part of the same consortium. Achieving full blockchain-enabled IT systems will require a significant overhaul of existing IT infrastructure, potentially incurring high costs [3]. To address this financial burden, hospitals, manufacturers, and other PMS stakeholders should collectively contribute to support this technological advancement. Several entities have already initiated investigations into the application of BCT, with the European Commission launching the EU Blockchain Observatory and Forum [74]. This initiative aims to monitor PMS data, analyze trends, and address emerging concerns. Additionally, it is part of a broader effort to establish a blockchain standard for the European Union, with the potential for future extension to the Eudamed database [74].

## Conclusions

BCT holds immense potential and offers significant synergy with the current needs of adopting PMS for medical devices. The successful implementation of our proposed solutions requires a solid understanding of the requirements and existing challenges within the medical device sector. It also necessitates continuous collaboration with a BCT specialist. The involvement of a BCT specialist is essential to ensuring that the new system operates effectively. Their expertise will eliminate the risk of the system failing due to developers lacking knowledge of how blockchain functions and its impact on the PMS process. This innovative approach has the potential to provide a more efficient means of managing medical device PMS, offering numerous benefits to the various stakeholders involved, including support for new regulatory initiatives.
